# Causal Links between Hypovitaminosis D and Dysregulation of the T Cell Connection of Immunity Associated with Obesity and Concomitant Pathologies

**DOI:** 10.3390/biomedicines9121750

**Published:** 2021-11-23

**Authors:** Natalia Todosenko, Maria Vulf, Kristina Yurova, Olga Khaziakhmatova, Larisa Mikhailova, Larisa Litvinova

**Affiliations:** 1 Center for Immunology and Cellular Biotechnology, Immanuel Kant Baltic Federal University, 236001 Kaliningrad, Russia; tod_89@mail.ru (N.T.); kristina_kofanova@mail.ru (K.Y.); hazik36@mail.ru (O.K.); larisalitvinova@yandex.ru (L.L.); 2 Department of Therapy Medical Institute, Immanuel Kant Baltic Federal University, 236016 Kaliningrad, Russia; mihalysa@mail.ru

**Keywords:** vitamin D, autoimmune disease, Th17 cells, type 2 diabetes mellitus, inflammation, obesity, NASH

## Abstract

Subclinical inflammation in morbid obesity is associated with immune activation and the development of concomitant diseases. Impaired immune homeostasis and immune cell dysregulation in adipose tissue are associated with phenotypic and functional changes in the pool of T lymphocytes and the development of chronic hypovitaminosis D. Low vitamin D levels in obesity lead to the activation, proliferation and production of pro-inflammatory mediators by T cells. Hypovitaminosis D is the cause of a decrease in the functional potential of regulatory and anti-inflammatory lymphocytes and the maintenance of the inflammatory response. The exact molecular genetic mechanisms of the effect of vitamin D on T lymphocytes have not been fully elucidated. Therefore, uncovering the functional role of T cells and their relationship to vitamin D homeostasis in the context of obesity development may contribute to the development of new pathogenetic methods for clinical prediction of the risk of metabolic, oncologic, autoimmune and infectious complications. The review presents the molecular genetic mechanisms of the effect of vitamin D on adipose tissue resident T lymphocytes and the characteristics of vitamin D receptor expression, and analyzes the phenotypic and functional characteristics of potentially pathogenic T lymphocytes in relation to the development of obesity and its associated complications.

## 1. Introduction

The pathogenesis of obesity is closely related to changes in the homeostasis of immune cells in the intestine, adipose tissue and liver [1]. A high-calorie diet induces the development of pathogenic strains of microorganisms in the gastrointestinal tract and the disruption of endothelial cell density with the development of metabolic endotoxemia [2,3]. Microbial dysbiosis leads to immune cell dysfunction and increases the body’s susceptibility to infections [4].

Obesity is associated with chronic indolent inflammation with localized, distinct foci of inflammation in visceral adipose tissue and liver [1]. Activated populations of CD4^+^ T cells are an important pathogenetic component of inflammatory diseases [5]. Obesity and type 2 diabetes mellitus have been found to be associated with a significant increase in Th1 and Th17 lymphocytes in visceral and subcutaneous adipose tissue (and a change in cellular composition depending on the location of the adipose tissue) against a background of a decrease in the number and functional properties of T regulatory (Treg) cells [6].

It should be noted that hyperplasia and hypertrophy of adipose tissue activate stress signals in adipocytes that contribute to increased secretion of free fatty acids and reactive oxygen species (ROS) [7]. Oxidative stress that occurs in obesity and autoimmune diseases (AIDs) induces activation of the mTORC1 pathway, which provides glucose uptake and aerobic glycolysis by modulating the transcription factor HIF-1α [8]. The mTORC1-HIF-1α pathway stabilizes and enhances the transcriptional activity of the master regulator of Th17 cell development and differentiation, RORyt, under hypoxic conditions [9].

Obesity is associated with increased death of adipose tissue cells, leading to an increase in extracellular ROS, which acts as an alarm (danger) signal [10]. ROS has been shown to regulate Th17 cell responses through the purinergic receptor P2X7 [11,12]. Increased expression of the P2X7 receptor in visceral and subcutaneous adipose tissue of individuals with metabolic syndrome confirms the role of adipose tissue in Th17 lymphocyte differentiation [12]. Th17 cells play a key role in the pathogenesis of severe AIDs, cardiovascular disease (CVD) and cancers. Additionally, it is likely that the link between obesity and its associated diseases is low serum vitamin D levels [13].

In the literature, low concentrations of 25(OH)D_3_ in peripheral blood are described in AIDs (multiple sclerosis, systemic sclerosis, rheumatoid arthritis, insulin-dependent diabetes, systemic lupus erythematosus, Hashimoto’s thyroiditis) [5,13,14,15], atherosclerosis, dyslipidemia, DM2, obesity, CVD, cancers and infections [16].

Vitamin D has been found to have a broad spectrum of functions. One of these is to act directly on immune system cells by regulating their proliferation and metabolism [17]. Activated T lymphocytes express the nuclear and cytoplasmic vitamin D receptor (VDR) [14]. Cytoplasmic ligation of VDR and transfer of the formed complex into the nucleus contribute to the expression of pro-inflammatory genes (sensitive to the action of vitamin D, namely the response elements of the vitamin D receptor regions (VDRE), CTLA-4, PLC-y1, IL-13, IFNy, IL-17A, IL-17F, IL-26) in T lymphocytes [13,18].

Interestingly, hypovitaminosis D induces the differentiation and development of Th17 cells [17,19]. Moreover, constant antigenic stimulation and a pro-inflammatory microenvironment against the background of chronic inflammation contribute to an accelerated formation of a memory T cell pool, which plays an important role in the pathogenesis of many serious diseases. Memory T cells formed in the long-term inflammatory process (e.g., in obesity) may acquire new functional properties.

Considering that Th17 lymphocytes may constitute a significant part of the memory T cell pool, the molecular processes involving vitamin D (hypovitaminosis), which affect their development, could serve as a fundamental basis for understanding the pathogenesis of non-infectious inflammatory conditions and for finding specific targets to prevent the formation of T cells with autoreactive properties. In addition, Th17/T memory cells may be a link between the state of vitamin D deficiency and the development of obesity, as well as biomarkers for assessing (the degree of) the risk of developing more severe complications.

In our opinion, the study of the functional role of memory T cells and their relationship with vitamin D in the context of obesity development has enormous potential and clinical significance for predicting the risk of metabolic, oncological and autoimmune complications.

## 2. Vitamin D Metabolism

**Figure 1:**
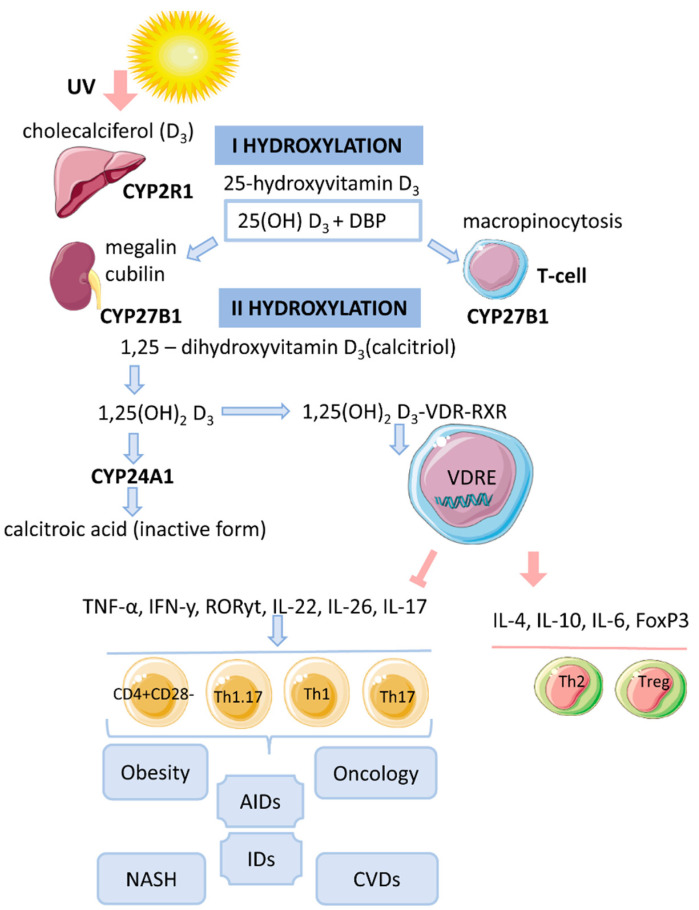
The mechanism of action of vitamin D on T cells in obesity.

Vitamin D is a fat-soluble secosteroid, 70–80% of which is synthesized in a photolysis reaction in the skin [20]. Under the influence of ultraviolet radiation (UV) from the B spectrum of the sun, the process of photosynthesis in the epidermis is activated. In this process, 7-dehydrocholesterol (provitamin D) is converted into previtamin D_3_, which undergoes isomerization of three double bonds after a thermochemical reaction, forming the inactive vitamin D3 (cholecalciferol). The synthesis of vitamin D_3_ takes up to 3 days from the time the skin is exposed to UV rays. This process is the main source of vitamin D and it depends on the intensity of UV radiation (latitude, season, time of day), skin pigmentation and air pollution [20,21,22] (Figure 1).

Low vitamin D levels can be partially compensated for by eating mushrooms, fortified dairy products, egg yolk and fatty fish (up to 20–30%). Vitamins D_2_ (ergocalciferol) and D_3_ from ingested food enter the small intestine, bind to chylomicrons (such as lipids) and are then transported to the lymphatic system and bloodstream [22,23]. The first hydroxylation of vitamin D at C-25 (carbon atom-25) occurs in the liver under the influence of 25-hydroxylase (CYP2R1), an enzyme of the cytochrome P-450 (CYP) family, forming 25-hydroxyvitamin D3 (or hydroxylated calcitriol 25(OH)D_3_) [24].

CYP27A1 and CYP2D25 are also reported to be enzymes responsible for the synthesis of 25(OH)D_3_. Considering that 25(OH)D_3_ is the major form of circulating vitamin D and its amount is proportional to synthesized and dietary vitamin D, it is the most reliable biomarker of circulating vitamin D levels. The circulation time of the inactive form of vitamin D-25(OH)D_3_ is 2–3 weeks [21] (Figure 1).

25(OH)D_3_ also forms a complex with a carrier protein, vitamin D binding protein (DBP), which is a plasma alpha-1 globulin. It is transported to the kidney where the filtration process takes place [25]. DBP has been shown to interact with vitamin D metabolites. It is also a ligand for a transmembrane protein of the kidney, megalin. DBP leads to the uptake of 25(OH)D_3_ by renal tubular epithelial cells via endocytosis [26]. The second surface receptor for DBP in renal proximal tubules is cubilin. Their combined action ensures the penetration of the inactive form of vitamin D into the cells [21].

Here, the second hydroxylation occurs mainly by the enzyme 1α-hydroxylase (mitochondrial CYP27B1) with the formation of calcitriol or an active biological hormone—1,25(OH)_2_D_3_ [27]. CYP27B1 consists of cytochrome P-450, ferrodoxin and ferrodoxin reductase [21]. The expression of CYP27B1 has also been detected in other organs such as the epidermis (skin), lymph nodes, colon, central nervous system, placenta, sweat glands, adrenal glands and immune cells [28].

The kidney also contains the enzyme 24-hydroxylase (CYP24A1), which converts 25(OH)D_3_ to 24,25(OH)_2_D_3_ by hydroxylation at C-24 (carbon 24) [29]. Furthermore, 1,25(OH)_2_D_3_ is converted to calcitroic acid, which is functionally inactive [20,25]. These toxic metabolites are excreted in the bile, feces and urine [20]. CYP24A1 is present in all VDR-expressing cells. It is believed that 1,25(OH)_2_D_3_ is the preferred substrate for CYP24A1 [21]. Thus, CYP24A1 can regulate the level of circulating active vitamin D and modulate the intracellular concentration of 1,25(OH)_2_D_3_ by stimulating the appropriate genomic responses.

## 3. Cytosolic Complex 1,25(OH)_2_D_3_-VDR-RXRF

The biological effect of 1,25(OH)_2_D_3_ is related to the activation of the cytosolic VDR receptor, whose active ligand is DBP and an indirect change in the transcriptional activity of vitamin D-associated genes [17]. VDR is a transcription factor and belongs to the family of nuclear receptors for steroid hormones (receptors for retinoic acid, thyroid hormones, sex hormones, adrenal hormones). The human VDR gene consists of eight coding exons (two non-coding) and two promoters and is located on chromosome 12 [21]. The VDR protein contains 427 amino acid residues. It acts as an obligate heterodimer that interacts with the retinoid X receptor (RXR), which subsequently causes the translocation of the complex into the nucleus and binds in a ligand-dependent manner to the promoter regions of target genes that are sensitive to vitamin D [17,20]. The functional domains of VDRs are the highly conserved NH2-terminal DNA-binding domain (DBD) and the more variable COOH-terminal ligand-binding domain (LBD). The DBD is a region with two zinc fingers, each containing a zinc atom in a tetrahedral arrangement with four invariant cysteine residues [21,30,31].

The lipophilic molecule 1,25(OH)_2_D_3_ can pass through the cell membrane and interact with the VDR [20]. Binding of 1,25(OH)_2_D_3_ to the VDR leads to conformational changes in the structure of the VDR protein that facilitate interaction with RXR and coregulatory complexes involved in the transcription of target genes [21]. Subsequently, the cytosolic complex 1,25(OH)_2_D_3_-VDR-RXR migrates to the nucleus, where it interacts with VDRE [14] (Figure 1).

In the nucleus, the 1,25(OH)_2_D_3_-VDR-RXR complex interacts with histone acetyltransferases, transcriptional coactivators, corepressors and chromatin-restructuring complexes to modulate the transcription of target genes [18].

## 4. Genomic Mechanism of Action of Vitamin D (T-lymphocytes)

After the identification of VDR in activated T lymphocytes, vitamin D has been proposed as a regulator of the immune system [13]. The active form of vitamin D, 25(OH)_2_D_3_, has an immunomodulatory effect on many components of the innate and adaptive immune systems [17]. 1,25(OH)_2_D_3_ regulates differentiation and maturation of subpopulations of innate immunity cells, antigen presentation and the production cytokines and chemokines. 1,25(OH)_2_D_3_ inhibits the inflammatory response by suppressing the expression of Toll-like receptors 2 and 4 (TLR2/4) and the secretion of pro-inflammatory mediators (IL-1, IL-6, TNF-α). In addition, the active form of vitamin D negatively regulates the differentiation, maturation and immunomodulatory capacity of dendritic cells (DCs) by reducing the expression of MHCII, CD40, CD80, CD86 and the maturation proteins CD1a, CD83 [17]. 1,25(OH)_2_D_3_ also inhibits the DC-mediated activation process of T cells and decreases the expression of inflammatory mediators (IL-12, IFNy in DCs) [5,20]. 1,25(OH)_2_D_3_ inhibits the proliferation of T lymphocytes and the production of IFNy and IL-17 and increases the secretion of IL-4 and IL-10. Thus, 1,25(OH)_2_D_3_ enhances the regulatory Th2 immune response and induces the differentiation of Treg cells, thereby reducing the pro-inflammatory potential of Th1 and Th17 cells.

The complex 1,25(OH)_2_D_3_-VDR-RXR blocks the formation of NFAT/AP-1. It is known that the formation of the NFAT/AP-1 complex is necessary for the activation of the IL-2 promoter.

The repressive effect of 1,25(OH)_2_D_3_ on IFNy gene transcription is due to the direct interaction of VDR-RXR with the silencer regions on the gene promoter. 1,25(OH)_2_D_3_ enhances the production of IL-4 by Th2 cells and potentiates the regulatory properties of Treg cells by activating the expression of the transcription factor FoxP3 [5,22].

The immunomodulatory effect of 1,25(OH)_2_D_3_ is also associated with an increase in IL-6 secretion, which may lead to a shift in the balance towards the Treg cell response [20,32].

1,25(OH)_2_D_3_ prevents IL-17 production by suppressing inflammation and Th17-mediated autoimmunity [33,34].

The mechanism of IL-17 suppression by 1,25(OH)_2_D_3_ is based on blocking NFAT binding to the IL-17 gene promoter, sequestration of Runx1 factor by the VDR, recruitment of histone deacetylase (HDAC) and induction of FoxP3 expression [22]. In addition, the VDR interacts with the P105/P50, P100/P52 and P65 proteins of the NF-kB factor.

Palmer et al. showed inhibition of the major Th17 transcription factor, RORyt, under the influence of 1,25(OH)_2_D_3_ [21].

In hypovitaminosis D, the addition of 1,25(OH)_2_D_3_ has been shown to help suppress the formation of inflammatory infiltrates and inhibit the expression of RORyt/IL-17 in spleen tissue of mice by preventing the translocation of P65 into the nucleus [35].

The stimulatory effect of 1,25(OH)_2_D_3_ on the production of anti-inflammatory cytokines (IL-4, IL-10) may be indirect and dependent on numerous intercellular contacts and the state of cell activation.

DCs are known to be an important target for 1,25(OH)_2_D_3_. Under the influence of vitamin D, their maturation is affected by transcription-mediated reprogramming of metabolic pathways (simultaneous increase in glycolysis and oxidative phosphorylation). Moreover, the mechanism for reducing IL-12 production in DCs involves binding of VDR-RXR to the NF-kB site on the IL-12p40 gene promoter. Thus, 1,25(OH)_2_D_3_ can alter T cell behavior by regulating DCs, causing T lymphocyte anergy [20] and converting pro-inflammatory Th1/Th17 cells into more tolerant cells (Th2/Treg cells) [21].

## 5. Vitamin D and T-lymphocytes

Expression of VDR has been detected in cells of innate and adaptive immunity [20]. Vitamin D exerts an immunoregulatory function by inhibiting proinflammatory cells and promoting the development of anti-inflammatory populations, thus participating in the processes of immune tolerance. Cells of the immune system express the enzyme CYP27B1 and can locally convert 25(OH)D_3_ to the active form 1,25(OH)_2_D_3_, where it is further utilized or locally released to neighboring cells [13,14]. However, the importance of the systemic circulating level of 25(OH)D_3_, which has a longer half-life, has been demonstrated [14].

The degree of activation of T lymphocytes is directly related to their phenotypic characteristics and functional potential. During their life (maturation), T cells (CD4^+^) undergo several stages of differentiation, starting with naïve (Tn) and then sequentially progressing into the group of effector cells (Teff) (including Th1, Th2, Th9, Th17, Th22 cells), central memory cells (Tcm)/memory effector cells (Tem) and terminally differentiated memory T cells (Temra) [36]. The level of VDR expression was found to be related to the activation of T lymphocyte; naive T cells are thus insensitive/resistant to vitamin D.

### 5.1. Th1 and Th2 Cells

Studies on obesity and DM2 have shown a significant increase in Th1 and Th17 cells and a decrease in the number and functional properties of Treg cells [6].

1,25(OH)2D3 was found to suppress IFNy production [18], blocking Th1 cell differentiation and development, against a background of increased secretion of IL-4 (which stimulates Th2 cell formation) [13].

### 5.2. Non-Pathogenic Th17 Cells and Treg

Obesity caused by a high-fat diet results in low secretion of Th17-associated cytokines in the intestine and adipose tissue, but high in the liver [37,38].

Non-pathogenic Th17 lymphocytes have a high intracellular content of polyunsaturated fatty acids and cholesterol esters and also a low content of saturated and monounsaturated fatty acids and triglycerides [39]. Melatonin, cholesterol, cholesterol sulfate and steroid lipids (oxysterols) have been shown to be ligands of RORα (RORyt) [40].

A distinctive feature of Th17 cells (compared to Treg cells) is the de novo synthesis of neutral fatty acids. The energy cost of this process in Th17 cells is compensated by glycolysis [41].

1,25(OH)_2_D_3_ has been shown to inhibit Th17 cell differentiation and stimulate Treg cell development [13]. 1,25(OH)_2_D_3_ inhibits the secretion of IL-17A, IL-17F, IL-22 and IL-26 [18].

### 5.3. Th1.17-Cells

Consumption of high-calorie foods leads to the disruption of immune cell homeostasis in the gastrointestinal tract and the formation of a pro-inflammatory pool of Th17 lymphocytes that can migrate to adipose tissue and liver. The development of an inflammatory response against a background of metabolic endotoxemia and adipocyte hypertrophy/hyperplasia can activate the plastic properties of Th17 cells, which, under similar conditions, can first transform into IL-17^+^IFNy^+^ lymphocytes and then into pathogenic Th17 cells (pTh17/Th1.17/nonclassical Th1) involved in the development of AIDs [42].

Th1.17 cells have been found to form a heterogeneous population that differs in phenotypic characteristics and cytokine-producing properties and depends on epigenetic stimuli that regulate permissive (H3K4me3) and repressive (H3K27me3) histone marks [32,43]. One of the characteristics of pTh17 lymphocytes is a high production of pro-inflammatory mediators: IFNy, TNF-α, GM-CSF and cytotoxic molecules (granzymes, perforin), together with a low secretion of IL-17, which is also reflected in the genotypic characteristics of these cells [44].

It was found that 1,25(OH)_2_D_3_ suppresses DC maturation and reduces the number of IL-17^+^IFNy^+^ cells during in vitro cultivation [45].

### 5.4. Memory T Cells

Vitamin D is an important regulator of CD4^+^ lymphocyte differentiation [13]. A link between vitamin D deficiency and an increase in the number of proinflammatory memory CD4^+^CD28 cells has been suggested [16]. In NASH, often associated with obesity, an increase in local infiltration of CD4^+^CD28 memory cells into the liver has been observed [46].

Loss of the co-stimulatory molecule CD27 (as well as CD28) by T cells (transition to the memory T cell pool) is associated with increased gene expression (mTORC1, ICC, cholesterol metabolism and glycolysis) [47].

This allows us to consider the population of Th1.17 cells that have undergone multiple antigenic stimulation as part of the memory T cell pool.

Autoreactive Th1.17 cells have been found to contribute significantly to the development of hypovitaminosis in AIDs [48]. This occurs through high expression of the enzyme CYP24A1 and inactivation of 1,25(OH)_2_D_3_. Interestingly, higher expression of CYP24A1 in autoreactive CD4^+^ T lymphocytes was found in men compared to women [49,50]. In addition, statins were found to induce the transcriptional activity of PPRA-y, which competes with VDR for interaction with the common ligand RXR. The latter represses the expression of the CYP24A1 gene and triggers the transcription of the CYP27B1 gene, thereby increasing local and systemic levels of 1,25(OH)_2_D_3_ [48].

Studies have shown a clear link between inflammation and vitamin D deficiency. Low 25(OH)D_3_ levels have been shown to be a risk factor for obesity, atherosclerosis, etc. [16]. In various AIDs and chronic inflammation, there is an accumulation of CD28-negative cells [46]. At the same time, hypovitaminosis D promotes an increase in the number of CD4^+^CD28^null^ cells, which acquire the cytotoxic cell phenotype and the ability to secrete IFNy, perforin and granzymes without activating costimulatory molecules [16]. At the same time, the addition of vitamin D to cultures of unstimulated (quiescent) T lymphocytes did not change the low expression of VDR [14].

CD28^null^ cells are newly activated memory/effector cells equipped with combinations of adhesion molecules and chemokine receptors that mediate the invasion of liver tissue. Upon reactivation, CD4^+^CD28^null^ cells secrete high levels of proinflammatory cytokines TNF-a and IFNy [46]. Furthermore, pathogenic memory T cells express a multidrug resistance receptor (MDR-1 or P-glycoprotein) on the cell membrane, similar to cancer stem cells that are resistant to chemotherapy. 1,25(OH)_2_D_3_ decreases the drug resistance of tumor cells [51]. Vitamin D may have a similar effect on pathogenic CD4^+^CD28^null^ cells in inflammatory diseases and AIDs.

Vitamin D has been found to decrease TNF-a production and to have a direct anti-inflammatory effect on CD4^+^CD28^null^ cells that accumulate in the liver in primary sclerosing cholangitis [46].

An important finding is the extent of involvement of memory T cells, including Th1.17 cells, in peripheral blood and inflammatory foci in AIDs. Circulating memory T cells are less committed and actively respond to the anti-inflammatory effects of 1,25(OH)_2_D_3_ and block IL-17 and IFNy. However, memory T cells localized in areas of active inflammation are the most highly committed Th1,17 cells (probably Temra) that are resistant to the action of 1,25(OH)_2_D_3_ [5].

Thus, the final affiliation of memory T cells to a particular phenotype plays a central role in attenuating the anti-inflammatory effects of vitamin D. VDR, as a transcription factor, regulates the expression of genes (IL-17,IFNy) containing functional vitamin D response elements (VDRE) [52,53]. This process is regulated by genomic variations and epigenetic mechanisms that lead to specific changes in 1,25(OH)_2_D_3_-mediated chromatin remodeling and VDRE availability [54].

Based on the above, variations in the response to 1,25(OH)_2_D_3_ depend on the activation state of T cells (naïve/activated) and the stage of differentiation (memory cells), as well as the degree of phenotypic affiliation (stability) of the cells, which is directly related to epigenetic changes that influence the resistance of tissue-specific T lymphocytes to the action of 1,25(OH)_2_D_3_.

## 6. Hypovitaminosis D in Obesity

Circulating 25(OH)D_3_/1,25(OH)_2_D_3_ is bound with DBP (85–90%) or albumin (10–15%) and only 1% of vitamin D is in free form. T lymphocytes take up DBP by macropinocytosis, although activated T cells express megalin. It has been found that the affinity of DBP for 25(OH)D_3_ is significantly higher than for 1,25(OH)_2_D_3_. In this case, DBP may also interact with actin and fatty acids. High fatty acid levels in obesity may exacerbate vitamin D deficiency due to competitive binding to the carrier protein.

Furthermore, oxidative stress against the background of an inflammatory response can lead to oxidative modification and carbonylation of proteins, resulting in an alteration of protein conformational structure and function. It has been shown that DBP carbonylation leads to a decrease in the anti-inflammatory effect of 25(OH)D_3_ on T lymphocytes [13]. On the one hand, under the conditions of oxidative stress and inflammation, this process leads to the local release of high concentrations of vitamin D to reduce the inflammatory response; on the other hand, the conformational changes of DBP during a chronic inflammatory response support vitamin D hypovitaminosis. Circulating levels of 25(OH)D_3_ are inversely proportional to plasma triglyceride and cholesterol levels [48].

Based on previously obtained data, theories have been formulated that partially explain the relationship between hypovitaminosis D and obesity [55,56,57].

(1) Volume dilution. Serum vitamin D levels decrease with increasing body size [58,59].
(2) Sequestration of vitamin D in adipose tissue. Vitamin D (synthesized by skin, supplements/medications) becomes tightly bound in fat stores and does not enter the bloodstream in sufficient quantity to maintain serum levels of 25(OH)D_3_ [55,57].
(3) Different ability to activate vitamin D in adipose tissue of lean and obese individuals [58]. In adipocytes, high expression of the enzymes 1α-hydroxylase (mitochondrial CYP27B1) and 25-hydroxylase CYP2J2 was found [55]. However, their expression is lower in obese people compared to lean people [60].

Moreover, vitamin D regulates the processes of lipogenesis and lipolysis in adipocytes. Against the background of vitamin D deficiency and a decrease in serum calcium levels, the production of parathyroid hormone increases [57]. This leads to an increase in intracellular calcium levels and activation of fatty acid synthase (FAS), promoting the process of lipogenesis in adipocytes [58].

The differentiation of preadipocytes into adipocytes (adipogenesis) is regulated by the enzyme lipoproteinase associated with 25(OH)D_3_ [57]. During adipogenesis, a number of signaling molecules are released, including WNT [58]. Hypovitaminosis D promotes adipogenesis by suppressing the WNT/b-catenin pathway [61]. Low concentrations of active vitamin D are unable to inhibit mRNA and phosphorylation of ERK (a signaling molecule of the MAPK pathway), thereby supporting the process of adipocyte differentiation.

In addition, vitamin D directly affects the expression of leptin (the satiety hormone). High leptin concentration indirectly inhibits the conversion of 25(OH)D3 to the active form and stimulates the production of FGF23 by osteoblasts/osteocytes [56]. However, FGF23 inhibits CYP27B1 in the kidney.

## 7. Molecular Mechanism of Action of Vitamin D on T Cells under Hypoxic Conditions Associated with Obesity

In obesity, the formation of local foci of hypoxia is observed. At the same time, hypoxia activates the transcription of the factor HIF-1α, which, together with mTORC1, promotes pTh17 cell differentiation [41]. Vitamin D has been shown to suppress LPS-induced expression of HIF-1α, thereby reducing the extent of hypoxia [62].

The mTORC1 pathway is believed to play an important role in the induction of plastic properties and the formation of Th1.17 cells (with a pathogenic phenotype) [63]. The PI3K (phosphatidylinositol 3-kinase)-AKT-mTORC1-S6K axis has been described as a positive regulator of Th17 cell differentiation that induces nuclear translocation of the RORyt factor [64].

In addition, hypoxia and other cellular perturbations (DNA damage, endoplasmic reticulum stress, energy stress) induce transcription of the gene DDIT4 (DNA Damage-Inducible Transcript 4 or REDD1). Recent studies have shown that binding of 1,25(OH)_2_D_3_ to VDR can increase gene expression of DDIT4. Considering that activation of DDIT4 leads to inhibition of mTORC1, their interaction could be regulated by 1,25(OH)_2_D_3_ [65].

In vitro studies of squamous cell carcinomas in mice (and humans) have shown an association between 1,25(OH)_2_D_3_ and mTORC1. The anticancer effect of 1,25(OH)_2_D_3_ has been linked to activation of the transcription of the DDIT4 gene, which acts as a molecular vector. 1,25(OH)_2_D_3_ inhibits the mTORC1 pathway [66].

In osteoblasts, the DDIT4 gene targets 1,25(OH)_2_D_3_ and inhibits cell proliferation [67]. High glucose levels inhibited the DDIT4 gene expression, while treatment of pancreatic β-cells with 1,25(OH)_2_D_3_ prevented the observed effect [68]. In the context of diabetic nephropathy (a common complication of type 1 and type 2 diabetes), hyperglycemia activates the mTOR pathway via PI3K/Akt and regulates cell growth and proliferation through direct phosphorylation (p70S6K, 4E-BP1). Simultaneously, 1,25(OH)_2_D_3_ induces the DDIT4 gene by blocking the mTOR signaling pathway [65]. Molitoris et al. (2011) showed that DDIT4 acts as an inhibitor of the mTOR pathway in thymocytes and stimulates the autophagy process (as a survival mechanism after dexamethasone exposure) [69]. It was found that the resistance of cells (acute lymphoblastic leukemia) to the action of glucocorticoids is probably related to impaired induction of the gene DDIT4 [70]. DDIT4 has been found to support optimal levels of T cell proliferation and survival [71].

At the same time, DDIT4 suppresses mTORC1 activity by inducing the TSC1/2 complex and regulates IL-17 production in patients with multiple sclerosis [72].

Thus, hypovitaminosis D might be directly related to low expression of the DDIT4 gene and activation of the mTORC1-HIF-1α pathway in conjunction with oxygen deprivation, leading to the formation/differentiation of pTh17 cells and maintenance (amplification) of a chronic inflammatory response.

Under pathological conditions, adenosine triphosphate (ATP) is released from intracellular stores into the extracellular space, where it acts as a stress signal (alarm, alert, DAMP) by binding to purinergic receptors. The purinergic receptor P2X7 (P2X7R), an extracellular ATP-dependent channel, is involved in the secretion of pro-inflammatory cytokines (which trigger an inflammatory response), cell death and autophagy [38,73,74].

Obesity is associated with increased extracellular ATP levels (as a result of the death of fat cells and other cells) [75]. At the same time, high release of ATP from cells in response to inflammatory mediators is the major mechanism for neutrophil activation and immune response [76]. Increased expression of the purinergic receptor P2X7 has been shown to promote the progression of inflammatory diseases [73]. Extracellular ATP enhances Th17 cell polarization through activation of surface receptor P2X7 in visceral adipose tissue [75]. At the same time, activation of DCs under the influence of ATP can create a favorable environment for macrophage maturation and pTh17 cell formation [77]. This is confirmed by an increase in the number of tissue-specific Th17 cells in metabolic syndrome.

CD39 ectonucleotides mediates the first step of the conversion (hydrolysis) of ATP to ADP and AMP to adenosine [38]. Activation of CD39 reduces the extracellular concentration of ATP and prevents the pro-inflammatory and pro-apoptotic effects induced by this nucleotide [74]. It is known that not only Treg cells express CD39. The presence of the CD39 molecule has also been demonstrated on Th17 cells in the inflamed areas of visceral adipose tissue [38]. Bai et al. (2014) reported that CD39 in combination with CD161 regulates Th17 cell proliferation through sphingomyelinase in patients with Crohn’s disease [78]. High expression of CD39 in Th17 lymphocytes was associated with low IL-17A production in patients with AIZ [79,80].

In a study of steroid-resistant patients with severe asthma, a positive role of 1,25(OH)_2_D_3_ in reducing the cytokine-producing potential of pathogenic IL-17^+^IL-22^+^ T cells in peripheral blood was demonstrated against a background of dexamethasone addition in vitro. The authors suggest that 1,25(OH)_2_D_3_ increases CD39 expression and probably restores the regulatory potential of Treg cells by suppressing IL-17 secretion. However, no clear mechanism of action of 1,25(OH)_2_D_3_ on Th17 cells was demonstrated in this work [81].

Therefore, a precise molecular mechanism of immune cell regulation under the influence of vitamin D is not known. However, a direct anti-inflammatory effect of this secosteroid has been demonstrated, aimed at modulating the properties of T cells through the activation of various signaling cascades.

## 8. Hypovitaminosis D in Obesity and Infectious Diseases

Infectious diseases are more severe when the body’s immune responses are impaired or weakened, which may be the case with chronic inflammatory processes, such as obesity. In addition, it can be argued that the association between low serum 25(OH)D levels and various diseases is due to a “reverse causality”, i.e., the disease state lowers vitamin D concentration proportionally to the severity of the disease [82]. Obesity is associated with low-grade systemic inflammation due to excess adipose tissue and vitamin deficiency, particularly hypovitaminosis D [83]. Vitamin D levels are inversely associated with respiratory tract infections [84].

Studies on the relationship between 25(OH)D concentration and COVID-19 severity have shown an inverse correlation [85]. It was found that the concentration of 25(OH)D was significantly lower in elderly patients with a positive COVID-19 PCR result than in patients with a negative result [86]. Obesity, diabetes and high body mass index (BMI) are risk factors for severe complications and poor prognosis in COVID-19 [83,87,88]. The total number of deaths from COVID-19 in the United States correlates significantly with the presence of obesity [87]. Obesity is known to bring damaging changes to the balance of gut microbiota and has also been linked to the prevalence of opportunistic microorganisms that affect Th17 cell pool formation [32]. Studies have demonstrated the presence of *Bacteroidetes, Firmicutes* and *Proteobacteria*, known as immune response-regulating microorganisms, in both lung and gut tissues [89]. Vitamin D deficiency, which occurs in obesity, supports dysregulation of the gut–lung axis and contributes to the body’s susceptibility to viral infections [90].

Middle East respiratory syndrome coronavirus (MERS-CoV), severe acute respiratory syndrome-related coronavirus (SARS-CoV) and SARS-CoV-2 are known to affect the lower respiratory tract [91]. SARS-CoV-2 is an enveloped RNA virus of the Coronaviridae family group 2b, encoding viral replicase and four structural proteins in the viral envelope (S spike, E envelope, M membrane) and nucleocapsid (N) [92]. SARS-CoV-2 enters the cell by interacting with the hydrophobic pocket of the extracellular catalytic domain of angiotensin-converting enzyme 2 (ACE2) [93]. After the virus enters the cell, ACE2 is suppressed, followed by a local increase in angiotensin levels II and the development of acute respiratory distress syndrome [94].

Type II pneumocytes intensively express ACE2 receptors and are the main target for coronaviruses [91,95]. COVID-19 disrupts the function of these cells and decreases the concentration of pulmonary surfactant (which prevents alveolar collapse) [96]. Surfactant contains protein A (SP-A), which binds to and neutralizes influenza A virus via sialic acid residues. 1,25(OH)2D has been shown to increase pulmonary surfactant production [82].

Notably, vitamin D decreases survival and inhibits viral replication by inducing antimicrobial peptides: cathelicidin and defensins [97].

Vitamin D also increases free ACE2 concentration [98], which competitively prevents SARS-CoV-2 from entering cells via the ACE2 receptor, thereby reducing lung injury [99]. Calcitriol induces the synthesis of α-1-antitrypsin, which is important for lung integrity and repair, by IL-10 produced in CD4^+^ T cells [82].

Generally, vitamin D increases the expression of ACE2 by regulating angiotensin II concentration and prevents blood vessel constriction in COVID-19 infected lungs [100]. In addition, vitamin D inhibits renin production, which positively regulates the formation of angiotensin II [101].

Due to its anti-inflammatory effect, vitamin D is able to reduce cytokine storm, which is linked to more severe COVID-19 and increased mortality [97,102,103].

Endothelial dysfunction has been shown to contribute to the development of vascular inflammation and coagulopathy mediated by COVID-19 [104]. Vitamin D is thought to reduce oxidative stress and activation of the NF-kB pathway, thereby preventing endothelial dysfunction [104,105,106].

Elevated serum levels of 25(OH)D have been shown to protect against acute respiratory infections [107]. In a laboratory study, 1,25(OH)D reduced rotavirus replication in vitro/and in vivo [97]. Clinical studies with vitamin D (4000 IU/per day) have confirmed a reduction in dengue virus infection [97]. Environmental studies show that increasing vitamin D serum concentrations with supplements reduces the risk of influenza infection in the winter period [83,90]. Finally, a meta-analysis confirmed the protective role of vitamin D high concentrations of (400–1000 IU for up to 12 months) against acute respiratory infections [108].

Because of those aforementioned, it is necessary to conduct large-scale clinical trials to optimize vitamin D use in the treatment of viral infections (especially COVID-19) in the context of obesity.

## 9. Conclusions

Hypovitaminosis D is a possible link between subclinical inflammation in obesity and the development of concomitant severe diseases and a decrease in the functional properties of immune cells. It is known that the development of many pathologies (including metabolic disorders) is accompanied by the formation of autoreactive/hyperactivated T lymphocytes with pathogenic and pro-inflammatory potential, which are in the final stages of differentiation and are characterized by unique properties.

Hyperplasia and hypertrophy of adipose tissue in obesity lead to increased secretion of free fatty acids and ROS [7], triggering the mTORC1-HIF-1α signaling cascade that stimulates Th17 cell development and differentiation [9].

Moreover, an enhanced cell death is accompanied by increased extracellular alarmin—ATP [10]. ATP regulates Th17 cell response via the purinergic receptor P2X7 in visceral and subcutaneous adipose tissue [11,12].

Nuclear and cytoplasmic expression of the vitamin D receptor in activated T lymphocytes mediates its immunometabolic effect [109,110].

At the same time, low levels of active vitamin D-1,25(OH)_2_D_3_ (calcitriol), oxidative stress, and ROS maintain inflammatory states and stimulate the development of pathogenic Th17 and T memory cells [109]. Also of interest are pathological inflammatory conditions in which the expression of VDR in T-lymphocytes is not disturbed. Still, upon ligation of the receptor, the "classical" anti-inflammatory effect is not observed [5,110].

The study of the functional role of memory T cells and their relationship with vitamin D has excellent potential and great clinical significance for predicting the risk of metabolic, oncologic, autoimmune and infectious complications associated with the development of morbid obesity. Further deciphering the molecular genetic mechanisms of vitamin D action on memory T cells (localized in adipose tissue), the specificities of vitamin D receptor expression and the phenotypic and functional characteristics of potentially pathogenic T lymphocytes in the context of obesity development and associated complications will allow the identification, on the basis of fundamental knowledge, of new targets for therapeutic treatment and enable the development of new approaches for diagnosing and reducing the risk of developing severe pathologies associated with the different phenotypes of morbid obesity (Figure 1).

## Data Availability

The data are available upon request from the author’s correspondents.
